# Efficacy and Safety of Silodosin and Dutasteride Combination Therapy in Acute Urinary Retention due to Benign Prostatic Hyperplasia: A Single-Arm Prospective Study

**DOI:** 10.1155/2016/4975851

**Published:** 2016-04-18

**Authors:** Kazuhisa Hagiwara, Takuya Koie, Hiromichi Iwamura, Atsushi Imai, Shingo Hatakeyama, Takahiro Yoneyama, Yasuhiro Hashimoto, Chikara Ohyama

**Affiliations:** Department of Urology, Hirosaki University Graduate School of Medicine, Hirosaki 036-8562, Japan

## Abstract

This study aimed to assess the efficacy of combination therapy with dutasteride and silodosin in patients with acute urinary retention (AUR) caused by benign prostatic hyperplasia (BPH). Eighty consecutive patients with a first episode of AUR were enrolled in this study. All patients received silodosin 8 mg and dutasteride 0.5 mg daily. Trial without catheter (TWOC) was attempted every 2 weeks until 12 weeks after the initiation of medication. The primary endpoint was the rate of catheter-free status at 12 weeks. Voided volume (VV), postvoid residual urine (PVR), uroflowmetry, International Prostatic Symptoms Score (IPSS), and quality of life due to urinary symptoms (IPSS-QOL) were also measured. All patients were followed up for more than 12 weeks and were included in this analysis. The success rate of TWOC at 12 weeks was 88.8%. VV and maximum urinary flow rate were significantly higher at 2, 4, 8, and 12 weeks compared with the time of AUR (*P* < 0.001). IPSS and IPSS-QOL were significantly lower at 2, 4, 8, and 12 weeks compared with the time of AUR (*P* < 0.001). In conclusion, a combination of dutasteride and silodosin therapy may be effective and safe for patients with AUR due to BPH.

## 1. Introduction

Acute urinary retention (AUR) is a common urological emergency and is defined as a sudden and painful inability to pass urine [1]. In most male patients, AUR is attributed to the natural history of benign prostatic hyperplasia (BPH) [2]. Baseline variables for AUR patients with BPH are old age, severe lower urinary tract symptoms (LUTS), low peak flow rate, increased postvoid residual urine (PVR), enlarged prostate, and high serum prostate-specific antigen (PSA) levels [3, 4].

Immediate management of AUR requires complete bladder decompression by inserting an indwelling urinary catheter. Although a trial without catheter (TWOC) is the standard of care for AUR [5], TWOC typically involves removing the catheter after 3 days, which allows only 23%–40% of patients to void successfully [5, 6]. TWOC in which catheter removal followed 2-3 days of *α*-blocker (AB) improves success rates [7]. Kumar et al. reported that silodosin significantly increased the chances of successful TWOC after AUR [8]. Alternatively, a 5*α*-reductase inhibitor (5-ARI) alone or combined with AB is a treatment option in patients with LUTS due to BPH [9]. Long-term combination therapy with AB and 5-ARI has been shown to reduce progression to AUR and the need for surgery, in addition to providing symptomatic relief [10, 11]. However, the utility of combination therapy with silodosin and dutasteride in TWOC after AUR has not been studied to date. The aim of this prospective single-arm study was to assess the impact of 8 mg silodosin twice daily and dutasteride 0.5 mg once daily on the outcome of TWOC after a first episode of AUR.

## 2. Materials and Methods

### 2.1. Patient Selection

The study protocol and informed consent documents were reviewed and approved by the Hirosaki University Institutional Review Board. All patients gave their written informed consent to participate in the trial.

All consecutive patients presenting with their first episode of spontaneous AUR were enrolled. Exclusion criteria were urinary tract infections, urological tumors, clot retention, urethral stricture, chronic urinary retention, urolithiasis, drug abuse, neurogenic lower urinary tract dysfunction, and a history of prostatic surgery or urological treatments.

Clinical details including medical history, date of catheterization, retention urine volume, digital rectal examination (DRE) findings, and prostate volume were recorded at the time of AUR. Voided volume (VV), PVR, uroflowmetry, LUTS (graded according to International Prostatic Symptoms Score (IPSS)), and quality of life due to urinary symptoms (IPSS-QOL) were also measured every 2 weeks. Serum PSA levels were measured at the time of AUR before catheterization and 12 weeks after the initiation of medication.

### 2.2. Treatment

After initial management of AUR with immediate bladder decompression by inserting an indwelling urinary catheter, all patients were given 4 mg oral silodosin tablet twice daily and a 0.5 mg dutasteride capsule once daily until catheter removal.

A TWOC was attempted every 2 weeks until 12 weeks after the initiation of medication. In this study, the catheter was removed after the instillation of 200 mL normal saline. The patients were considered to have achieved a catheter-free status (CFS) if they could void voluntarily with a PVR of <100 mL and did not require recatheterization in the next 24 h.

### 2.3. Endpoints and Statistical Analysis

The primary endpoint was the proportion of successful TWOCs. The secondary endpoints were changes over time in IPSS score and PVR. Other outcomes measured were the cumulative incidence of invasive treatments related to BPH, including transurethral resection of the prostate and stenting and changes over time in the serum PSA levels and prostate volume.

Data were analyzed using SPSS 22 statistical software (IBM Corp., Armonk, NY, USA). Statistical comparisons were made using the chi-square test for qualitative variables and Student's *t*-test for quantitative variables. The influence of study variables on the TWOC success rate was tested using logistic regression methods. All *P* values were 2-sided, and the significance level was set at <0.05.

## 3. Results

In total, 80 patients presenting with a painful AUR between September 2010 and June 2013 from public (50%) or private (50%) healthcare practices were enrolled. All the patients completed the trial protocol. The clinical characteristics of the enrolled patients are listed in Table 1. All patients completed the trial protocol and experienced no adverse event.

**Figure 1:**
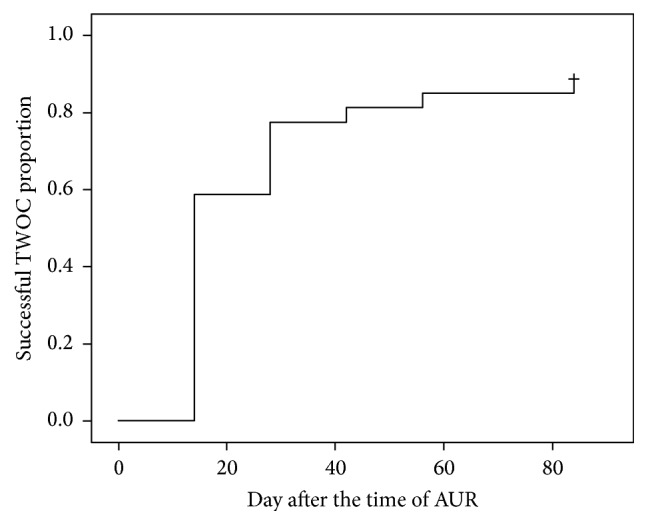
Kaplan-Meier estimate of successful trial without catheter for patients with acute urinary retention. The success rate of trial without catheter was 88.8% at 12 weeks.

The success rate of TWOC was 88.8% at 12 weeks (Figure 1). Nine patients (11.2%) had an indwelling catheter owing to voiding failure. Of 71 patients with successful TWOC, 7 (9.9%) had a second episode of AUR within the first 3 months after the successful TWOC. Eight (10%) patients, including 4 with a successful TWOC, required BPH surgery.

**Figure 2:**
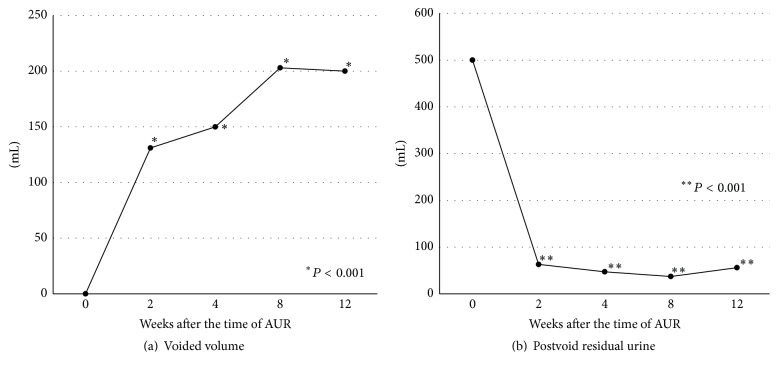
The chronological changes in voided volume (a) and postvoid residual urine (b). (a) Voided volume gradually increased throughout the entire evaluation period and was significantly higher at 2, 4, 8, and 12 weeks than that at the time of acute urinary retention (^*∗*^ *P* < 0.001). (b) Postvoid residual urine at 2 weeks was 63 mL and significantly lower at 2, 4, 8, and 12 weeks than that at the time of acute urinary retention (^*∗∗*^ *P* < 0.001).

**Figure 3:**
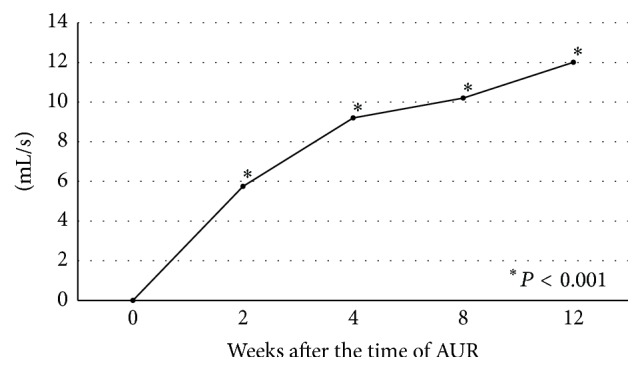
Chronological changes in maximum urinary flow rate. Maximum urinary flow rate was significantly higher at 2, 4, 8, and 12 weeks than that at the time of acute urinary retention (^*∗*^ *P* < 0.001).

Regarding VV, PVR, and uroflowmetry, 35 patients were evaluated at 2 weeks, 33 patients at 4 weeks, 10 patients at 8 weeks, and 36 patients at 12 weeks. The chronological changes in VV and PVR are shown in Figure 2. VV gradually increased throughout the entire evaluation period and was significantly higher at 2, 4, 8, and 12 weeks compared with the time of AUR (^*∗*^ *P* < 0.001; Figure 2(a)). The median PVR at 2 weeks was 63 mL (interquartile rate (IQR) 14–63), which was maintained throughout the entire evaluation period (^*∗*^ *P* < 0.001; Figure 2(b)). The maximum urinary flow rate (Qmax) also gradually increased throughout (Figure 3). Qmax was significantly higher at 2, 4, 8, and 12 weeks compared with the time of AUR (^*∗*^ *P* < 0.001).

**Figure 4:**
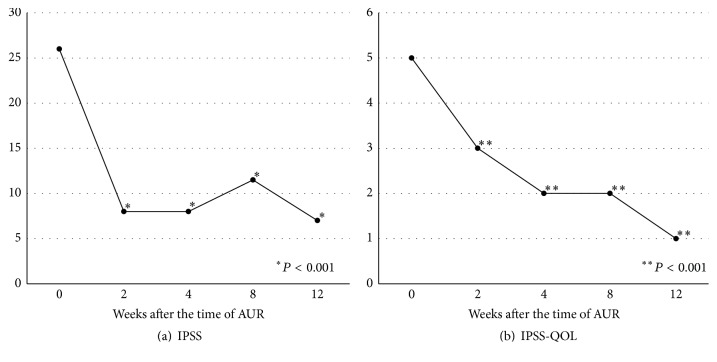
Chronological changes in International Prostate Symptom Score (a) and quality of life due to urinary symptoms (b). (a) International Prostate Symptom Score was significantly improved at 2, 4, 8, and 12 weeks compared with that at the time of acute urinary retention (^*∗*^ *P* < 0.001). (b) Quality of life due to urinary symptoms was significantly improved at 2, 4, 8, and 12 weeks compared with that at the time of acute urinary retention (^*∗∗*^ *P* < 0.001).

The chronological changes in IPSS and IPSS-QOL are shown in Figure 4. IPSS at 2 weeks was 8 (IQR 7–9), which was maintained throughout the entire evaluation period (^*∗*^ *P* < 0.001; Figure 4(a)). IPSS-QOL gradually decreased throughout the entire evaluation period and was significantly lower at 2, 4, 8, and 12 weeks compared with the time of AUR (^*∗∗*^ *P* < 0.001; Figure 4(b)).

The median serum PSA level was 5.8 ng/mL at the time of AUR and 3.3 ng/mL at 12 weeks. Median prostate volume was 46.4 mL at the time of AUR and 38.1 mL at 12 weeks. Serum PSA level (*P* = 0.003) and prostate volume (*P* < 0.001) were significantly decreased at 12 weeks compared with the time of AUR.

## 4. Discussion

AUR is a common urological emergency in men. The immediate treatment of AUR is catheterization followed by TWOC after a variable interval. The management of AUR is not standardized because of a lack of existing guidelines, and important differences exist among institutions and countries with regard to the duration of catheterization and management of TWOC. Until recently, standard management in patients with AUR was prostatic surgery within a few days or weeks after a first AUR episode. However, patients who underwent AUR-related emergency prostatectomy with a urinary catheter were at a greater risk of peri- and postoperative complications, including sepsis or death, compared with those who underwent elective prostatectomy for symptoms alone [12]. The relative risk was 1.8 for perioperative complications, 1.6 for postoperative complications, and 3.3 overall for hospital death, with a relative risk of 26.6 at 30 days and 4.4 at 90 days [12]. In addition, Murray et al. reported that up to 23% patients with AUR did not require prostatectomy based on urodynamic assessment [13]. In contrast, TWOC involves removing the catheter after 1–3 days, which allows 23%–40% of patients to void successfully [5, 6].

ABs are recommended as first-line treatment for LUTS with moderate to severe symptoms due to BPH [14]. Currently, TWOC after AB therapy is also the recommended treatment option for patients with AUR. The large randomized, double-blind, placebo-controlled alfuzosin in another AUR study investigated the impact of AB on the outcome of TWOC [7]. Three hundred sixty patients with a first episode of AUR-related BPH were randomized to receive 10 mg alfuzosin once daily or placebo for 2-3 days following catheterization [7]. The successful voiding rate was significantly higher in patients treated with alfuzosin than in those treated with placebo (62% versus 48%, resp.; *P* = 0.002) [7]. Various clinical trials have demonstrated that ABs, including tamsulosin and alfuzosin, are effective and safe in AUR management, with successful removal percentages that range from 48% to 70% [15]. Kumar et al. reported that patients receiving silodosin were twice as likely to void successfully as those receiving placebo [8]. The success rate of TWOC in the silodosin group was slightly better than those reported after tamsulosin or alfuzosin in TWOC [6, 7, 15]. One reason for this may be that silodosin's *α*-1A : *α*-1B binding ratio is extremely high (162 : 1), leading to its selective action in the lower urinary tract with minimal side effects on blood pressure regulation [8]. Therefore, silodosin has a good uroselectivity compared with other ABs and may have better efficacy than tamsulosin [16, 17].

Several reports suggested that the post-AUR use of AB prevents recurrence and increases the success rate of self-voiding [7, 18]. However, despite continuing medication, 17.1% of patients required surgical treatment during 6 months [18]. AUR patients with large prostate volume were most at risk for recurrent AUR or prostatic surgery [3]. Recently, combination therapy with 5-ARI and AB provided significantly greater benefit than either monotherapy for various outcomes in patients with LUTS due to BPH and prostatic enlargement [10, 11]. The Medical Therapy of Prostatic Symptoms study showed (with a mean follow-up period of 4.5 years) that combination of finasteride and doxazosin therapy significantly reduced the risk of overall clinical progression of BPH, AUR, and need for invasive therapy in patients with LUTS due to BPH [10]. McConnell et al. also reported that the reduction in the risk of AUR and the need for invasive therapy throughout the study may be attributed to a reduction in prostate size [10]. According to the combination of Avodart® and tamsulosin study, dutasteride and tamsulosin combination therapy significantly reduced the relative risk of AUR- or BPH-related surgery over 4 years by 66% compared with tamsulosin monotherapy [11]. In addition, combination therapy significantly decreased the relative risk of clinical progression and symptom deterioration in patients with IPSS ≥4 points [11]. These data suggest that combination of dutasteride and tamsulosin therapy in patients with LUTS due to BPH provided rapid and durable symptom benefit and reduced the long-term risk of BPH clinical progression [11].

Our findings were limited because this was a nonrandomized single-arm study with a relatively small sample size. In this study, the success rate of TWOC was 88.8% at 12 weeks and achieved relatively higher CFS than previous reports [7, 8, 15]; furthermore, relatively few patients with a successful TWOC required surgical treatment compared with those in previous reports [7]. In addition, Qmax, IPSS, and IPSS-QOL were significantly improved and remained favorable throughout the 12-week study.

These results suggest that combination therapy with dutasteride and silodosin could have potential to achieve a relatively high success rate of TWOC. A prospective randomized trial is necessary to determine whether the combination therapy is superior to single-agent therapy.

## Figures and Tables

**Table 1:** Clinical characteristics of the study participants.

Age (years)	
Median (IQR)	75.0 (68.8–81.3)
Retention volume (mL)	
Median (IQR)	500 (375–1000)
IPSS	
Median (IQR)	26 (16–31)
IPSS-QOL	
Median (IQR)	5 (5-6)
PSA (ng/mL)	
Median (IQR)	5.80 (3.15–12.70)
Prostate volume (mL)	
Median (IQR)	46.4 (34.9–69.6)

IQR, interquartile range; IPSS, International Prostatic Symptoms Score; IPSS-QOL, quality of life due to urinary symptoms; PSA, prostate-specific antigen.
